# The Role of IgG4 in Autoimmunity and Rheumatic Diseases

**DOI:** 10.3389/fimmu.2021.787422

**Published:** 2022-01-25

**Authors:** Maria Maslinska, Joanna Dmowska-Chalaba, Michal Jakubaszek

**Affiliations:** Early Arthritis Clinic, National Institute of Geriatrics, Rheumatology and Rehabilitation, Warsaw, Poland

**Keywords:** immunoglobulin G4, rheumatic diseases, autoimmune diseases, malignancy, allergic diseases

## Abstract

The distinguishing of the IgG4-related disease (IgG4-RD) from among other rheumatic diseases has brought attention to the IgG4 subclass of immunoglobulins. It is the least numerous subclass among immunoglobulins G. In general, IgG4 is considered to be non-inflammatory and tolerance inducing, due to its unique structure. However, in IgG4-RD this antibody plays a pathogenic role in activation of the fibrinogenesis and of the inflammatory process; there are also suggestions that it may be a marker of an abnormal inflammatory response. The importance of IgG4 for the pathogenesis of allergic diseases, with a vital role of its ratio to immunoglobulin E (IgE/IgG4 ratio), has been known for years. The role of IgG4 in the course and pathogenesis of rheumatic diseases is still being researched and is not yet fully understood. Increased IgG4 levels have been revealed in rheumatoid arthritis, although no clear link between this phenomenon and disease activity has been demonstrated. There are articles on the potential importance of IgG4 concentration (of both elevated and decreased serum levels) in Sjogren’s syndrome. Additionally, anti-nuclear IgG4 antibody significant titers have been detected in SLE patients, and it has been suggested that the effect of these antibodies on complement consumption and the production of proinflammatory cytokines may play a role in inhibiting the progression of SLE. IgG4 plays a role in autoimmune diseases other than rheumatic diseases, such as pemphigus, bullous pemphigoid, idiopathic membranous glomerulonephritis, or myasthenia gravis, but also in helmints infections. Research shows the importance of IgG4 in malignancy of neoplasms. Melanoma cells are known to stimulate IgG4 production through a modified Th2-based inflammatory response. The role of this immunoglobulin in cholangiocarcinoma is also considered as possible. The aim of this review article is to discuss the current knowledge of IgG4 not only from the perspective of the IgG4-RD but also from a point of view of other autoimmune diseases with particular emphasis on rheumatic diseases.

## Introduction

IgG4 is a subclass of immunoglobulin G. Its role in the inflammation is still being defined, as the significance of its anti-inflammatory activity and tolerance-inducing properties is counteracted by its pathogenic features present in recently identified IgG4-dependent diseases. IgG4 is the least numerous of four IgG subclasses and accounts for only about 5% of IgG immunoglobulins in the human body. Its unique properties, dissimilar to other IgGs, are the lack of influence on the classical complement component pathway and, through a property called “fab-arm exchange” (FAE), inhibition of the formation of large immune complexes (1).

These specific qualities of IgG4 are related to its varied affinity to specific Fc gamma receptors (FcγRs); in fact, IgG4 has no affinity to the receptors FcγRIIIa and FcγRIIIb (1, 2).

IgG4 may mimic rheumatoid factor (RF) activity by interacting with other immunoglobulins G, although IgG4 binds to their constant domains, while a classical RF acts *via* variable domains (3, 4). Zack et al. (3) also found that in rheumatoid arthritis (RA) RF IgG represents mainly the IgG4 class.

IgG4 blocking of the binding of IgG1 to C1q and thus inhibiting the activity of IgG1 underlie anti-inflammatory properties of IgG4 (5, 6).

Over the years, these properties of IgG4 have been analyzed, revealing its vital role in a number of autoimmune diseases, including pemphigus, bullous pemphigoid, idiopathic membranous glomerulonephritis, or myasthenia gravis, as well as its association with parasitic infections (7). The importance of IgG4 in the course of some neoplasms, e.g., cholangiocarcinoma or melanoma, was also noted (8). However, it was the establishment of IgG4-related (IgG4-RD) diseases that caused a wider interest in this subclass of immunoglobulins among scientists, immunologists and clinicians of many specialties. The aim of this article is to discuss, from the clinical point of view, the currently known importance of IgG4, especially in rheumatic diseases.

## Structural and Physiological Difference of IgG4 From Other Immunoglobulins G

Immunoglobulins G are important in secondary immune response, particularly in response during infections and allergy. Four subclasses of immunoglobulin G have different abilities and affinity to type of FcR receptor, as well as the possibilities for activating the complement pathways.

**Figure 1:**
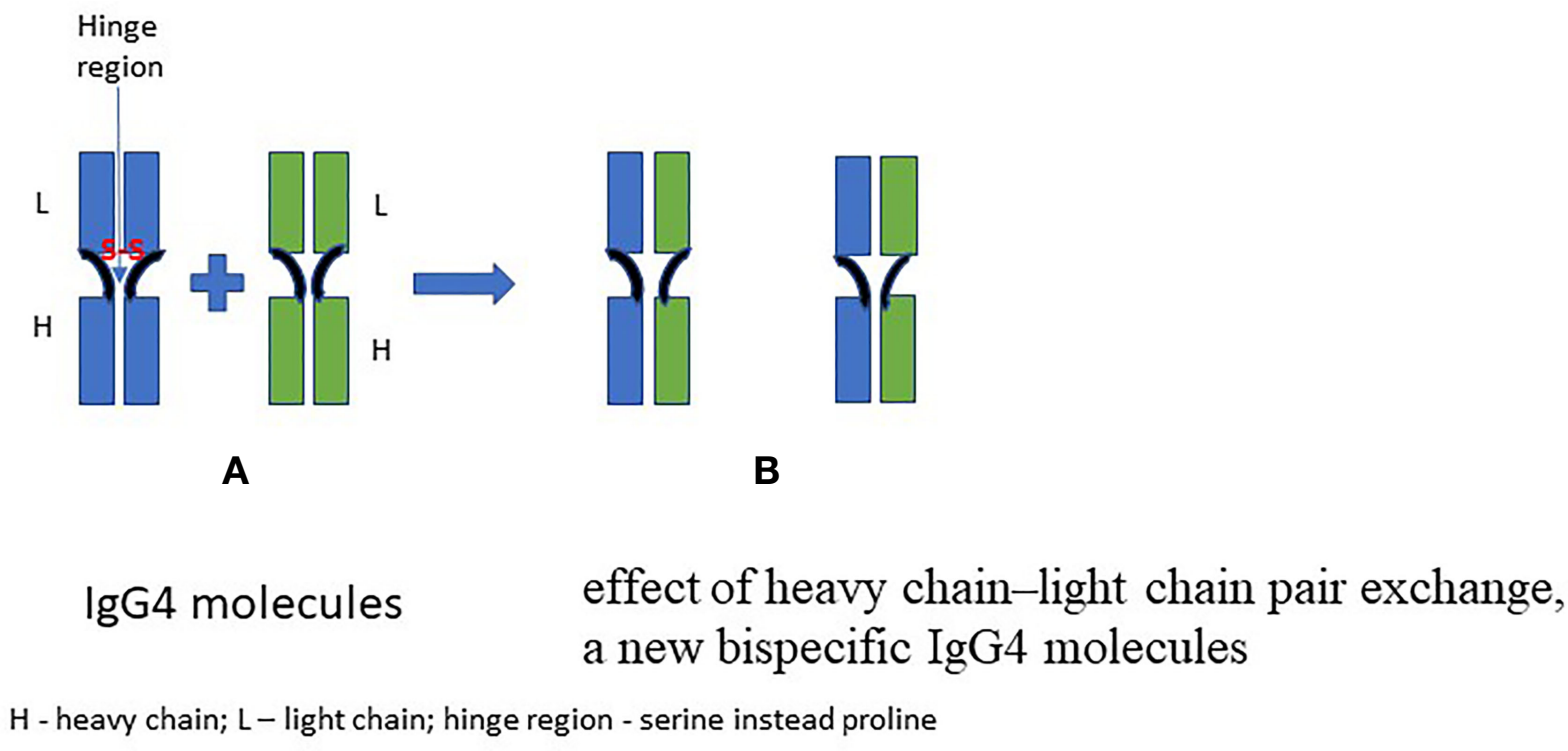
A schematic presentation of the FAE phenomenon (6, 9).

Focusing on IgG4, its molecule is composed of two heavy chains (with three specific constant domains: CH1, CH2, CH3, and one variable VH domain) and two light chains (consisting of two domains: one constant—CL and one variable—VL). The antigen-binding region of IgG4 consists of both constant (CL) and variable (VL) domains of the light chain and the heavy chain, a variable domain of the heavy chain (VL) and one of its constant domains (CH1). The IgG molecule is bonded by disulfide bridges inside the heavy and light chains and between heavy chains in the hinge region. IgG4 can dissociate into two halves, each consisting of one heavy and one light chain. Such semi-molecules are able to join corresponding semi-molecules deriving from other IgG4—this unique property being called “Fab-arm exchange” (FAE). A bispecific antibody with two antigen-binding sites is thus generated. This mechanism is possible due to the replacement of proline for a serine at position 331 (“P331”; binding of the protein C1q) and a proline for a serine in the hinge region at position 228 (P228S) compared to IgG (9, 10). The FAE phenomenon is still not fully understood, as it has not been unequivocally established how this phenomenon is being regulated. It has been observed that glutathione (GSH) can initiate FAE *in vitro*, while *in vivo* it can be caused by certain drugs (e.g., natalizumab, humanized monoclonal antibody against alpha-4 (α4) integrin) and it can run spontaneously (6). **Figure 1** shows the general principle of the FAE.

This phenomenon causes high variability of IgG4 that generally prevents it from forming immune complexes under chronic antigen stimulation, which possibly plays a role in inhibiting inflammatory reactions. However, the formation of immune complexes with IgG4 seems likely under certain conditions—this phenomenon being described in RA, IgG4-RD, and membranous nephropathy (11, 12). Its occurrence is attributed to the fact that the Fc region of IgG4 may react with other IgG through the Fc–Fc interaction. This phenomenon is not fully understood, and it is possible only in a solid phase of other immunoglobulins.

Apart from FAE mechanism, IgG4 does not have the ability of bivalent antigen binding, suggesting that it should not play an important role in the context of inducing autoimmunity. However, IgG4 is associated with various autoimmune diseases in which it is thought to have diverse range of functions—as it is discussed further below.

Immunoglobulins G bind FcγRs by its crystallizable fragment Fc; FcγRs and these receptors are on most effector cells of the immune system such as monocytes, macrophages, NK cells, mast cells, eosinophils, neutrophils, basophils, dendritic cells, and platelets (13, 14). These receptors are also responsible for various cell responses (activation and/or inhibition) and interactions in the immune system. In **Table 1**, the overview of the main types and roles of FcγRs and its affinity of IgGs especially IgG4 are presented.

**Table 1:** The main types and roles of FcγRs and its affinity of IgGs (13–17).

Fcγ receptor/subclasses binding	Cells expression	Role	IgG4 affinity
FcγRI IgG1, IgG3, IgG4	Monocytes, macrophages, dendritic cells, neutrophils, mast cells	Activation	**
FcγRIIaH IgG1,/IgG2/, IgG3, IgG4 FcγRIIaR IgG1,/IgG2/, IgG3, IgG4	Neutrophils, monocytes, macrophages, dendritic cells, basophils, eosinophils	Activation/inhibition	** **
FcγRIIb/c IgG1,/IgG2/, IgG3, IgG4	B cells, dendritic cells, mast cells, basophils/NK cells, monocytes, macrophages, neutrophils	Inhibition/activation	*
FcγRIIIaF IgG1, IgG2, IgG3, FcγRIIIaV IgG1, IgG2, IgG3, IgG4	NK cells/monocytes, platelets, macrophages	Activation/inhibition	**-** **
FcγRIIIb IgG1, IgG3	Neutrophils, eosinophils, basophils	Activation	**-**
FcγRn (bind its ligand in pH <6) IgG1,/IgG2/, IgG3, IgG4	Monocytes, macrophages, neutrophils, dendritic cells, endothelial cells, epithelial cells	Recycling transport uptake	***

-no affinity; **moderate affinity; ***strong affinity.

IgG4 presents affinity to FcγRI, FcγRIIa, and FcγRIIIav, low affinity to FcγRIIb/c, and no affinity to FcγRIIIaF and FcγRIIIb receptors. All immunoglobulins G, including IgG4, present high affinity to the FcγRn receptor. This receptor is responsible for the transport of IgG across the intestinal mucosa, placenta, and mammary gland, and due to this activity, the balance of IgG and albumin in the body remains stable. These functions of FcγRn depend on the intracellular signal transduction and activation caused by the combination of its extracellular domain and the IgG Fc domain (15, 16). As it has no affinity to FcγRIIIb and has low affinity to C1q, it cannot activate the classic complement pathway. It also inhibits the binding of C1q to IgG1, thus inhibiting the activity of this immunoglobulin. IgG4 is considered as a “blocking antibody” due to its ability to compete for the same epitopes with other immunoglobulins, e.g., IgE, thus preventing IgE-dependent allergic responses. The lack of ability to form immune complexes does not activate the classical complement cascade, so it does not stimulate antigen presentation.

The regulation of IgG4 production is closely associated with that of IgG1. The production of both these immunoglobulins is stimulated by Th2 lymphocytes *via* release of cytokines (IL- 4, IL-13) in the course of chronic exposure to the antigen (10–13). What is interesting is that IL-10, which stimulates IgG4 secretion, is produced not only by regulatory T lymphocytes but also by regulatory B lymphocytes, which themselves produce IgG4. This mechanism plays an important role in the immune tolerance and anti-inflammatory effect of IgG4 (10, 18).

To consider clinical effects of IgG4 presence, it is necessary to point out its properties, which could underlie the role it may play in certain diseases.

The main features of IgG4 antibodies are summarized in **Table 2**.

**Table 2:** Immunoglobulin G4 main characteristic (2, 11).

Immunoglobulin G4	
Basic features	Molecular mass 146 kDa Half-life of IgG4 molecule—21 days Serum abundance maximum 5% of total IgG Placenta transfer
Immunological features	High affinity to receptor FcγRI Lower affinity to FcγRII Response to proteins (++), polysaccharides (+/-) No response to allergens No complement component 1q (C1q) binding Immunologically inert Functionally monovalent
Serum concentration	Elevation in chronic exposure to the antigen Elevation in immune tolerance state Elevation in chronic inflammatory process (also in asymptomatic infection)

Diseases, in which IgG4 involvement is of clinical significance, can be basically divided into three groups: those in which it has a pathogenic role, those in which its effects are considered to be protective and finally a group in which the role of IgG4 is still debatable and needs a further investigation. **Table 3** presents a list of diseases or main clinical problems and the role assigned to IgG4 in them (1, 4, 10).

**Table 3:** The role of IgG4 (1, 4, 10).

Role	Protective	Pathogenic	Not fully understood
Disease	Allergic (inhibition of hypersensitivity) − Beekeepers − Laboratory workers − Allergen specific immunotherapy	IgG-4 ARD − MuSK-myasthenia gravis − Pemphigus foliaceus − Pemphigus vulgaris − Thrombotic thrombocytopenic purpura	IgG-4—RD See **Table 4**
	Helmints infection (inhibition of IgE dependent hypersensitivity reactions)	Helmints infection (persistence of infection) Cancer e.g., melanoma, cholangiocarcinoma (disadvantageous suppression)	Rheumatoid arthritis

IgG4-ARD, autoimmune related diseases; IgG4 RD, IgG4-related diseases.

## IgG4 Autoimmune-Related Diseases

In the majority of autoimmune diseases, a type III hypersensitivity is involved, in which circulating IgG, IgM, and IgA bind soluble antigen and may create immune complexes. Complement activation and activation of immune cells (Fc receptors) cause a cellular cytotoxicity, opsonization, phagocytosis, cell damage, and inflammatory process. As it has been described above, the unique structure of IgG4 causes that this antibody is an exception from this pathophysiological picture. Still, this does not rule out this antibody from being a causative agent of some autoimmune diseases. There are autoimmune diseases, in which autoantibodies consisted mainly of the IgG4 subclass of immunoglobulins G (IgG4-AID). These include diseases such as pemphigus vulgaris and foliaceus (19), thrombotic thrombocytopenic purpura (TTP) (20), muscle-specific kinase myasthenia gravis (MuSK-MG) (21), primary membranous nephropathy (22), Goodpasture syndrome (23), and chronic inflammatory demyelinated polyradiculoneuropathy (CIDP) (24). A number of autoantigens have been confirmed as a target for IgG4 antibodies; for example, in CIDIP the autoantigen is neurofascin-155 (NF155), with anti-NF155 IgG4 antibodies which were found in this disease (25, 26).

The diagnosis of IgG4-AID is based on the presence of antigen-specific autoantibodies with combination of specific clinical symptoms. In contrast to this, the diagnosis of IgG4-related diseases (IgG4-RD) is based on the presence of elevated serum concentrations of IgG4, pseudotumors, infiltrations of IgG4-positive plasma cells, fibrosis, and obliterative phlebitis (27).

In the 50’s, Witebsky postulated (28) that to identify an autoimmune disease three conditions have to be met: 1) recognition of an autoimmune response (autoantibody or cell-mediated), 2) identification of a corresponding autoantigen, and 3) induction of an analogous autoimmune response in experimental models, causing diseases in experimental animals. IgG4 ARD can be divided into three classes, based on the role IgG4 plays in the processes responsible for meeting Witebsky’s conditions. Class I includes diseases, in which two or all three Witebsky’s conditions are met. The pathogenicity of IgG4 antibodies can be proved by the transfer of these antibodies and their pathogenic action *in vitro* (28, 29). Class II includes disease, in which there is only indirect evidence of the pathogenicity of antibodies, e.g., the correlation of the IgG4 concentration with the severity of the disease or of the IgG4 presence with the occurrence of a specific phenomenon in the course of the disease. Class III diseases are those in which the role of IgG4 antibodies is not yet proven and their significance for the pathologic process has not been excluded (29).

In MuSK-MG, pemphigus foliaceus and vulgaris, thrombotic thrombocytopenic purpura (TTP), and chronic inflammatory demyelinating polyradiculoneuropathy (CIDP), it was concluded that IgG4 is involved in the processes responsible for meeting at least two of Witebski’s condition (27).

Less certain, although not devoid of evidence, is the effect of this antibody on the development and severity of diseases such as membranous nephropathy (proteinuria and nephrotic syndrome with immune deposits predominantly with IgG4) and AIDP (acute idiopathic demyelinating polyneuropathy) as well as anti-LGI1 autoimmune encephalitis and Morvan’s syndrome (peripheral nerve hyperexcitability, autonomic instability, encephalopathy). In this group, IgG4 takes part in mechanisms, which result in meeting first of Witebsky’s conditions (28, 29).

The weakest data link IgG4 with, e.g., such rare diseases as Goodpasture syndrome (antibodies against type IV collagen) or Iglon 5 parasomnia (Iglon-5 Ag). In this group, there is a proof of a presence of IgG4 autoantibodies, but there is no proof of their involvement in the pathological process (10).

What matters in the context of the topic under discussion in autoimmune diseases of the central and peripheral nervous system with importance of CASPR2, CASPR1, LGI1, or neurofascin 155 antibodies is that the shared feature is the predominance of antigen-specific antibodies of the IgG4 subclass (30, 31).

What can be seen are the main areas of the IgG4 ARD which are the skin, kidneys, and the central nervous and peripheral systems.

## IgG-4-Related Diseases

The IgG4-related diseases form a still growing group of fibroinflammatory diseases with special clinical features such as pseudotumors, storiform fibrosis, obliterative phlebitis, and organ damage. Their main histopathological feature is a presence of infiltrations by IgG4-producing plasma cells and eosinophils.

Apart from the above described diagnostic criteria, there has been suggestion by Hubers et al. (32) that the presence of A11-specific IgG4 antibodies might play a role of a specific marker for IgG4-RD. Their formation attenuated IgG1-mediated pro-inflammatory autoreactivity against annexin A11 in patients with IgG4-RD (32). This result is contrary to what might be assumed in an inflammatory disease, such as IgG4-RD, and may confirm the potentially anti-inflammatory role of IgG4 in IgG-RD.

### Storiform Fibrosis

The inflammatory infiltrates which include IgG4-positive plasma cells probably activate myofibroblasts and lead to a storiform fibrosis, characteristic for IgG4-RD. It can be assumed that the inflammatory process with the activation of B cells and activation of fibrosis (myofibroblasts) may proceed simultaneously. Changes in the histopathological image can also be expected over time—from the predominance of cellular infiltration to a weakly cellular image with dominant fibrosis (33). Della-Torre et al. (34) found that B cells from IgG4-RD patients produced the pro-fibrotic molecule PDGF-B and stimulated collagen production by fibroblasts. These B cells also expressed enzymes such as LOXL2, which are involved in extracellular matrix remodeling. Also, these authors presented that B cells produced chemotactic factors CCL-4, CCL-5, and CCL-11 and induced the production of these same chemokines by activated fibroblasts. In a cited work, the authors concluded that plasmablasts expressed intrinsic pro-fibrotic properties (34).

### Obliterative Phlebitis and Arteritis

In IgG4-RD, obliteration of venous vessels with their inflammation, i.e., lymphoplasmacytic infiltration of their walls, is found. The occlusion of these vessels without inflammation in the vascular wall is not a clue for the diagnosis of IgG4-RD (35). Arteries are less prone to inflammation in IgG4-RD. Their inflammation, when it occurs, is not necrotic, which is important in the differential diagnosis. Arteritis may be accompanied by retroperitoneal fibrosis, lung, and heart involvement or AIP.

According to the classification criteria for IgG4-RD, the ratio of IgG4-positive plasma cells to total IgG plasma cells should be over 40% (27). The group includes autoimmune pancreatitis (AIP), IgG4 kidney disease, or IgG4-related retroperitoneum fibrosis (Ormond’s disease). The updates of the American College of Rheumatology (ACR) and the European League Against Rheumatism (Eular) classification criteria, which were simultaneously published in 2020, take into account entry criteria (“characteristic clinical or radiologic involvement of a typical organ, e.g., pancreas, salivary glands, bile ducts, orbits, kidney, lung, aorta, retroperitoneum, pachymeninges, or thyroid gland [Riedel’s thyroiditis] OR pathologic evidence of an inflammatory process accompanied by a lymphoplasmacytic infiltrate of uncertain etiology in one of these same organs”) and exclusion criteria. The presence of the specific features has assigned certain point scores to it. If the entry criteria are met, no exclusion criterion is present and the total point score is ≥20 the Ig-G4 RD may be diagnosed.

Recent publications suggest candidates for potential new biomarkers in Ig-G4 RD, such as serum and tissue IgG2 or soluble IL-2 receptor (sIL2R)—for inflammation intensity and cc–chemokine ligand 18 (CCL18) for fibrosis (36). The IgG4+ plasmablast level was also highlighted as a potential biomarker useful for diagnosis, assessment during the course of the disease, and efficacy of treatment (37, 38). It has been demonstrated that the overexpression of TLR-7 in IgG4-RD stimulates macrophages 2 to produce IL-33 which in turn stimulates Th2 response (39), which suggests that the TLR-7 level might be also considered as a biomarker of the activation of Th2 pathway and of inflammatory response.

It was found that in AIP associated with the IgG4-RD, the serum concentration of laminin 511-E8 was significantly higher in comparison with healthy controls (40). Another study revealed a similar phenomenon concerning serum galectin-3 (41). What is important is that galectin-3 plays a significant role in the fibrosis (42) in IgG4-RD, as well as in an idiopathic pulmonary fibrosis (IPF) (43). The novel small-molecule TD139, an inhibitor of Gal-3, is currently investigated in I/II phase clinical trials in the treatment of patients with IPF (43).

The introduction of a concept of IgG-4 RD means that some diseases were treated until now as one uniform entity, such as autoimmune pancreatitis (AIP); a subtype concerning IgG-4 involvement was distinguished. In case of AIP, AIP-1 is a subtype regarded as IgG-4 RD. AIP 1 is characterized by lymphoplasmacytic sclerosing pancreatitis (LPSP) in histopathological assessment, with storiform fibrosis, IgG4-positive plasma cell infiltrations, and presence of other Ig-G4 RD features, including extraintestitial symptoms (44). In case of some diseases, such as the extraperitoneal fibrosis idiopathic type (Ormond’s disease), no official IgG4-RD subtype was distinguished, with both IgG4-RD and disease with no involvement of IgG4 having the same clinical picture (44–46). The primary Sjogren’s syndrome (pSS)—an autoimmune and inflammatory disease in which B-lymphocyte hyperreactivity, enlargement of salivary glands, the presence of autoantibodies, and mononuclear cell infiltration are the predominant features—can be confused with IgG4 RD. As it turns out, some cases initially recognized as a former Mikulicz’s disease, which is a pSS with a chronic salivary enlargement, without antibodies to ribonucleoproteins most characteristic for pSS (47)—such as anti-Sjögren’s-syndrome-related antigen A autoantibodies (anti SSA/RoAb) and anti-Sjögren’s syndrome-related antigen B antibodies (anti-SSB/La)—are now recognized as instances of IgG4-related disease (IgG4-related plasmacytic exocrinopathy) (48). The SSA/Ro antigen is a ribonucleoprotein complex containing hY-RNAs and two proteins differing molecular mass—Ro 52 and Ro 60 kD. The SSB/La antigen consists of protein of 48-kD mass (48). The Ro protein has been identified as an E3 ligase, which regulates negatively cytokine production induced by the IFNγ pathway, while the La protein works as a transcription termination factor of the RNA polymerase III transcripts (49).

The history of the discovery of IgG4 RD begun since the first description of the association of the elevated serum concentration of IgG4 with clinical features of autoimmune pancreatitis (AIP), by Hamano et al. in 2001. These observations and conclusions were confirmed and extended by other researchers primarily from Japan (50, 51). However, as mentioned above after analysis of clinical features of IgG4 RD (52), it was found that the image assigned to Sjogren’s syndrome, described by Polish physician Jan Mikulicz Radecki in 1888, also corresponds to an IgG4-dependent disease. Currently, the list of IgG4-RD is still being extended; it is presented in **Table 4**.

**Table 4:** The list of IgG4-RD (52–55).

Organ/system	Disease	Comments
Pancreas	Autoimmune pancreatitis	AIP type 1
Liver	IgG4 RD hepatitis	Hepatic mass, icterus
Bill ducts	IgG4-RD cholangitis	Often accompanied by AIP
Salivary glands	Mikulicz’s disease Kuttner’s tumor	Tumor, pain, swelling, mouth dryness Attention: differential diagnosis of Sjogren’s syndrome
Lungs	Pseudotumor, interstitial pneumonia, or pleuritis	Dyspnea, cough, hemoptysis, pleural effusion, deterioration of exercise tolerance, hypoxia (decrease in oxygen saturation)
Retroperitoneum	Retroperitoneal fibrosis	2 types Ureter obliteration Hydronephrosis Leg edema
Mesentery	Sclerosing mesenteritis	Uncharacteristic complaints—pain, flatulence Changes in CT imaging
Organ of sight	Orbital pseudotumor, chronic sclerosing dacryoadenitis, eosinophilic angiocentric fibrosis, idiopathic orbital inflammation	Pain,
Thyroid	Riedel’s thyroiditis	Asymptomatic or hypothyroidism, dyspnea, dysphagia, dysphonia neck pain,
Endocrine system other than thyroid glands	Hypophysitis	Pituitary inflammatory disorder with pituitary dysfunction (anterior and/or posterior) with diabetes insipidus (DI) and/or other endocrinopathy development.
Kidneys	Tubulointerstitial nephritis	Proteinuria, hematuria, hypocomplementemia, increased creatinine (chronic or acute renal failure)
Vessels	Aortitis, periaortitis, abdominal aneurism	May be associated with retroperitoneal fibrosis
Heart	Cardiac muscle infiltration/cardiac mass	Intra-cardiac mass may be asymptomatic; on auscultation heart murmurs due to an intra-cardiac obstructing mass, sinoatrial disturbances requiring pacemaker
Nervous system	Related hypophysitis, IgG4-related pachymeningitis	Headache, symptoms of spinal compression, radiculopathies
Prostate and male genitalia	Prostatitis, orchitis	Pain, pollakisuria, benign prostatic hyperplasia
Skin	Primary: Cutaneous plasmacytosis, pseudolymphoma, and angiolymphoid hyperplasia with eosinophilia Mikulicz disease (inflammation of lacrimal and salivary glands) Secondary: Psoriasis-like lesions Unspecified maculopapular or erythematous lesions Hypergammaglobulinemia, purpura, and urticarial vasculitis Ischemic digit	Primary: Histopathology: Marked lymphocyte and plasmacyte infiltration, IgG4+/IgG+ >40% No of IgG4+ cells per high-power field >10. primary Secondary: Plasmacyte infiltration with IgG4+/IgG+ >40% and/or perivascular IgG4 deposition

**Figure 2:**
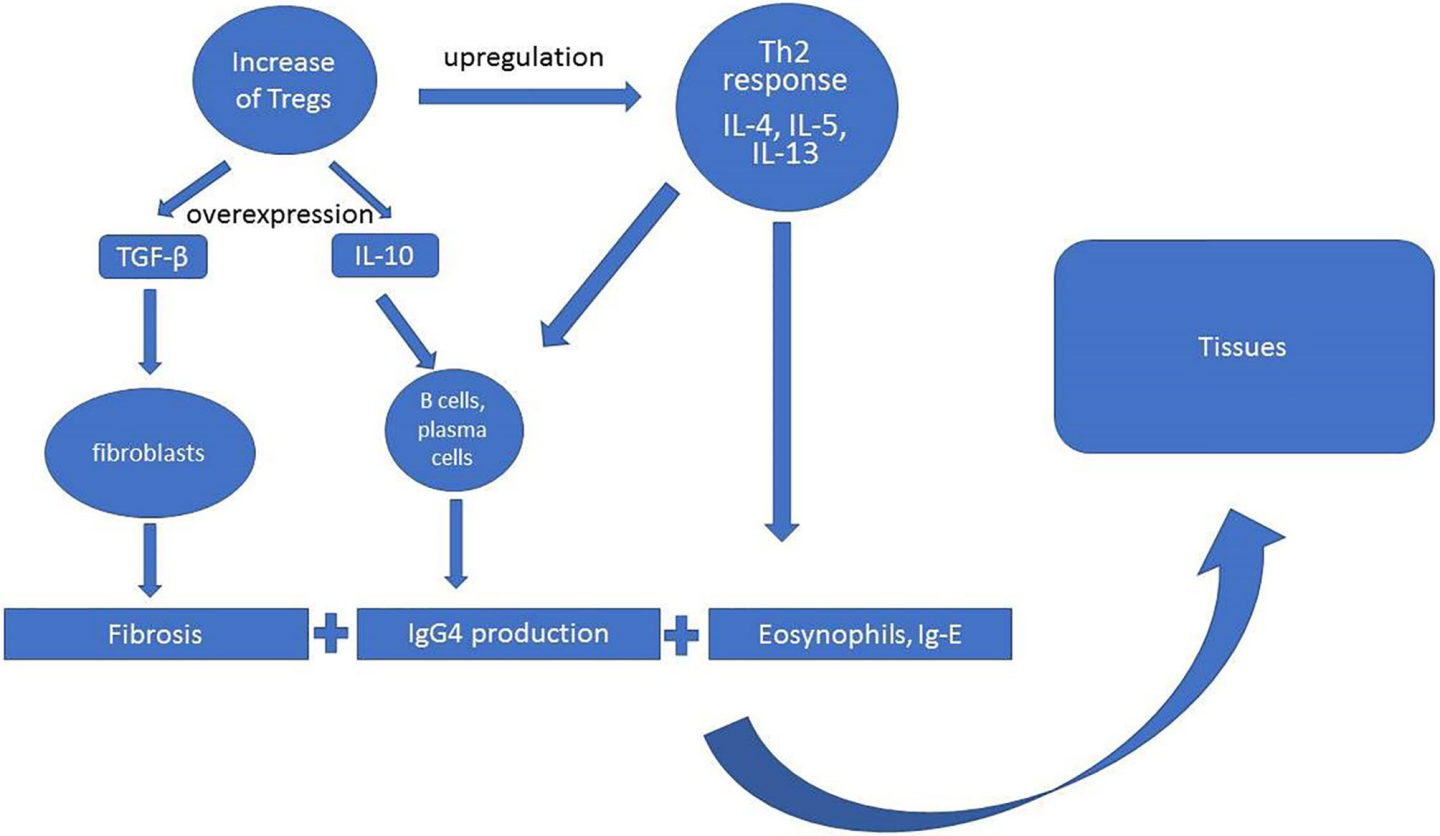
Outline of IgG4-related disease pathogenesis (55, 58, 59).

The scheme of the pathogenesis of IgG4-RD assumes the increased activity of T regulatory cells (Treg) with overexpression of IL-10, transforming growth factor β (TGF-β) production, and upregulation of Th2 response, in which predominant roles play interleukins 4, 5, and 13 (IL-4, IL-5, Il-13) (**Figure 2**). The activity of these cytokines translates into specific pathophysiological phenomena such as fibrosis (TGF-β), activation of B cells and plasma cells to IgG4 production (IL-10, IL-4, IL-5), and eosinophilia (Il-5, Il-4, Il-13) (30, 38). However, current ACR/EULAR classification criteria for IgG4-RD peripheral eosinophilia above 3,000/µl are considered as exclusion criteria depending on the clinical picture (27).

Presently, the knowledge of IgG4-RD is still expanding and the list of these diseases is constantly being supplemented. Several questions concerning IgG4-RD still remain to be answered, such as the following:

- is the presence of an increased amount of IgG4 in the patients’ sera a result of the activation of their production as an anti-inflammatory antibody in response to other factors, e.g., environmental such as infections, trauma, and other inflammatory processes (e.g., the inflammatory theory of atherosclerosis)? (56, 57)

- does the possibility of IgG4 complement activation other than the classic pathways (the mannose binding lectin pathway) better explain the role of this antibody in IgG4-RD?

- does IgG4 play a pathogenic role in IgG4-RD or is its increased concentration only an epiphenomenon related to other immunological processes taking place in IgG4-RD?

- does the effectiveness of therapies directed against B cells (anti-CD-20 and anti-CD-19) in IgG4-RD derive from the results they have on the IgG4 overproduction or from other effects of the depletion of B cell populations?

## Other Rheumatic Diseases and IgG4

### Rheumatoid Arthritis

In recent years, the importance of the IgG4 subclass in RA has been raised. There was an increase in the concentration of this immunoglobulin in RA patients compared to the control group. Among antibodies to cyclic citrullinated peptides (ACPA), IgG4 levels exceeded IgG2 and IgG3, and among immunoglobulins G belonging to the class of rheumatoid factor (RF), IgG4 levels were second only to IgG1. It is assumed that the persistent autoimmune stimulation in the course of active RA gives a boost to the production of IgG4 (60, 61). This observation was confirmed, as Yu et al. (62) found in 30.3% of studied RA patients (n= 433) elevated serum levels of IgG4. The infiltration of IgG+ plasma cells in IgG4 RD is a crucial feature of this disease, but was also described in synovium in the course of RA (63). Some reports suggest that the serum level of IgG4 ACPA may serve as a biomarker for monitoring the response of RA patients to therapy (61). Engelman et al. (64) demonstrated a significant decrease in IgG4 ACPA levels in responders to treatment, regardless of the treatment type (methotrexate as well as biologics). Carbone et al. (65) showed that blocking IL-6 by tocilizumab also reduces IgG4 anti-CCP antibodies, but such an effect was not shown on IgG1 anti-CCP antibodies. This may indicate the influence of IL-6 on the production of IgG4 anti-CCP antibodies in RA.

### Sjögren’s Syndrome

Primary Sjögren’s syndrome (pSS) is a chronic autoimmune disease in which under certain conditions, the tolerance is broken and B cells are stimulated depending on and independently of the activity of T lymphocytes, which causes their hyperreactivity and the production of antibodies mainly for ribonucleoproteins: anti-SSA (Ro52 + Ro60) and SSB/La. A further consequence of these events is the accumulation of mononuclear cells in infiltrates in the exocrine glands, which leads to their damage and clinical symptoms, such as dryness (changes in the secretion of, among others, tears, pancreatic juice, saliva) (66). This disease may be mild, but it can also be severe with involvement of organs and systems, including vasculitis and nerve damage. Phenomena such as hypocomplementemia, deficiencies of erythrocytes, platelets, lymphocytes, and the presence of cryoglobulins with cryoglobulinemic purpura are some of the possible immunological deviations in its course. Inflammation or enlargement of the salivary glands is quite common in pSS, with or without symptoms of dryness. Over the years, also another entity with a similar course, i.e., Mikulicz’s disease, was treated as a subgroup of patients with Sjögren’s syndrome. It is currently known, that in fact its discoverer Jan Mikulicz Radecki dealt with IgG4 RD (67). In IgG4 RD, sialadenitis and dacryoadenitis may also be a manifestation of this disease and in some studies in these patients the serum IFN-γ/IL-4 level was higher than in those with pSS (48).

There are contradictory reports on IgG4 in pSS; an increased concentration of this immunoglobulin in serum was reported in some of pSS patients. Mavgrani et al. (68) noted that this group had some features of IgG4-dependent disease. Another study shows a decrease in serum IgG4 concentrations in pSS patients, especially in those with high immune activity and C4 hypocomplementemia (69).

### ANCA-Associated Vasculitis

There are reports about increased IgG4 levels in some vasculitides, especially in eosinophilic granulomatosis with polyangiitis (EGPA)—formerly known as Churg-Strauss syndrome—and in granulomatosis with polyangiitis (GPA), associated with anti-neutrophil cytoplasmic antibodies (c-ANCA).

In EGPA with eosinophilia and other allergic features such as asthma, allergic rhinitis, and sinusitis, the increase in IgG4 levels can be explained as being analogous to allergic diseases. However, misdiagnoses of IgG4 RD are also possible. EGPA represents necrotizing vasculitis affecting medium-sized small vessels, while in IgG4-RD an involvement of larger vessels, such as the aorta, is expected. In case of doubts in the diagnosis, these diseases will be differentiated mainly by histopathology. The presence of ANCA antibodies may also point against IgG4-RD (70). Differentiation of GPA and IgG4-RD may be more difficult, and biopsy may not always bring unambiguous results. In a biopsy, especially from the orbit or sinus area, IgG4-positive plasmatic cells may be present in the GPA (71). The main distractor is the presence of ANCA antibodies in GPA. Increased serum IgG4 level is also seen in some cases of vasculitis; however, the IgG4/total IgG ratio is generally higher in IgG4-RD (72). In some cases, there is also the possibility of the IgG4-RD-vasculitis overlapping syndrome. Interestingly in GPA, IgG4-ANCA antibodies were detected, which have the ability to activate primed neutrophils (73).

### Systemic Lupus Erythematosus

The function of IgG4 antibodies in systemic lupus erythematosus (SLE) is still being discussed. Significant anti-nuclear IgG4 antibody titers (ANA-IgG4) have been detected in SLE patients, and it has been suggested that the effect of these antibodies on complement consumption and the production of proinflammatory cytokines may play a role in inhibiting the progression of SLE. The presence of anti-dsDNA IgG4 antibodies was also reported and was associated with skin lesions and kidney involvement in patients with SLE (74). Pan et al. (75) demonstrated in an *in vitro* constructed study using IgG4 healthy controls and IgG4 antibodies from SLE patients that the latter can inhibit complement consumption by the activity of autoantibody–autoantigen immune complexes (ICs). The authors concluded that SLE IgG4 inhibited the activation of the alternative pathway by other subclasses of autoreactive IgGs (75).

### Helmints Infection

The helmints infection is a dynamic pathophysiological state. At the start of the infection, Th1 response dominates. At later stages, Th1/Th2 responses become more balanced, while in chronic condition Th2 response with eosinophilia and increased concentration of IgG4 and Il-10 dominates. In the chronic infection, the downregulation of Th1-cell, Th17 cell response, and amplification of Th2 cell response occurs. Therefore, due to the activity of the abovementioned cytokines, the increased secretion of IgG4 can also be expected. Indeed, in parasitic infections, increased concentrations of IgG4 and IgE are found. A strong anti-parasite IgE response is associated with the resistance to infection (1, 3). The role of IgG4 in helmints infection is still analyzed. Essentially, two possible mechanisms are proposed that can lead to the inhibition of IgE-mediated immunity and resistance to helmints (74, 76). One is associated with the inhibition of a switch in plasma cell production from IgM *via* IgG4 to IgE. In this case, the inhibition of that switch at the IgG4 level disturbs the ratio of IgG4 to IgE in favor of the former. The second proposed mechanism assumes a competition between IgG4 (with low affinity for Fcγ receptors) and IgE for epitopes on the helmints surface or in a close environment. The similar situation is described in reactions to allergens. “Blocking antibody” activity explains the finding that IgE-mediated hypersensitivity reactions are rare in patients with chronic parasitic infections (76).

### Allergic Diseases

Allergic diseases as well as IgG4-RD have the same immunological features including predominant Th2 response with elevated IgG4 and IgE and eosinophilia. In allergic diseases, the balance between IgG4 and IgE depends on the activity of Th2 cells and of IL-4 and IL-10. Interleukin-4 stimulates the production of both IgG4 and IgE, while IL-10 only of IgG4. The higher the concentration of allergen-specific IgG4, the greater the tolerance to the allergen and the greater the inhibition of the allergic reaction. Hence, the immune tolerance state should be characterized by a high IgG4-to-IgE ratio (IgG4/IgE). As more often the ratio of IgE to IgG4 (IgE/IgG4) is used, its lower value indicates higher tolerance to the allergen (77, 78).

The ability of IgG4 to compete with IgE for the same epitopes in this case prevents the IgE-dependent acute reaction with degranulation of mast cells and release of histamine and bradykinin. In IgG4-RD, the presence of allergy, atopy, eosinophilia, and increased serum levels of IgE and IgE-positive mast cells in lymphoid, biliary, and pancreatic tissues is quite often described (79). Higher serum levels of IgE in IgG4-RD may indicate a relapse and, together with the increased concentration of IgG4, can be taken into account as a predictive factor (78). However, Della Torre et al. (80) presented different results of investigation concerning atopy in IgG4-RD patients. This study revealed that the majority of patients were non-atopic; atopy was confirmed in 33% of patients. The authors concluded that the prevalence of atopy in this group was similar to that of the general population. This indicates that this issue requires further investigation.

In allergic diseases, specific immunotherapy (SIT) relies on the administration of allergen extracts to produce the emergence of tolerance to those allergens. This method implies an impact on Treg cells and switching of allergen-specific B-cells toward IgG4 production (81). This causes an induction of Tregs, reduction in specific IgE levels, induction of IgG4 (blocking) antibodies, and shift from Th2 to Th1 response.

## Malignancies and IgG4

The IgG4 serum level may be elevated in IgG4-RD but may also be within a normal range—the latter does not exclude the diagnosis of IgG4-RD. On the other hand, the elevation of IgG4 may be observed in case of malignancy (82).

It is not uncommon that a pseudotumor lesion in an organ is treated as neoplastic, when in fact it is IgG4-RD. Therefore, in the differential diagnosis, IgG4-RD as a cancer mimicker should be taken under consideration. In 2.6% of patients with suspected pancreatic cancer, in whom the surgery was performed, AIP was a final diagnosis (83). In both clinical situations, biopsy and histopathological examination are essential for proper diagnosis and treatment (82).

In some malignancies, tumor cells escape host control, by inducing the switch of the production of autoantibodies toward IgG4, thus taking the advantage of the protective properties of IgG4. Such a phenomenon has been demonstrated for a number of neoplasms, including melanoma, extrahepatic cholangiocarcinoma, pancreatic cancer, or glioblastoma (55). The knowledge about cancer immunology is constantly growing. It has been found that in melanoma or cholangiocarcinoma, the concentration of tumor-specific IgG4 increases and the tumor cells stimulate their production. Tumor modifies the Th2 response and the activity of cytokines, especially IL-4 and IL-10. The increase in the activity of IgG4 with IgG1-inhibiting properties thus inhibits the antitumor effect of IgG1 and the activation of macrophages and in this way inhibits the death of the tumor cell (1, 26).

In a Chinese study presented in 2020, researchers found increased incidence of malignancy in patients with IgG4-RD. The authors pointed to risk factors, such as autoimmune pancreatitis, but also highlighted potential protective role of other phenomena, such as eosinophilia (8). Also, other studies reported malignancies in IgG4-RD patients; however, opposite conclusions are being presented as well. Hirano et al. (84) tested 113 of IgG4-RD patients and concluded that IgG4-RD is not associated with an increased incidence of total malignancies with comparison to the general population.

Another interesting conclusion presented by Wallace et al. (85) is that patients with IgG4-RD more often have a history of malignancy prior to their diagnosis. The most common malignancy in this studied group was prostate cancer and lymphoma. The authors suggested that the treatment of malignancy may also be a risk factor to the development of IgG4-RD or there are similar risk factors for both clinical situations.

An interesting research and conclusions were presented by Zhou et al. (86) in a study of 60 patients diagnosed with IgG4. The study revealed that as many as 60% (n = 38) were initially suspected of cancer, which resulted in greater interventions in 14 patients. General symptoms (e.g., fever, weight loss, fatigue), the presence of pseudotumors/infiltrations in various areas such as the abdominal cavity, salivary glands, or pleura, presence of lymphadenopathy, and increased inflammatory activity in laboratory tests led to the diagnostics being focused on proliferative processes. Oncological vigilance is important in any case with similar symptoms, but taking into account the possibility of the presence of an IgG4-related disease is necessary in order to avoid unnecessary burdensome procedures and inappropriate treatment. The simultaneous occurrence of IgG4 disease and a malignancy is also most commonly with lymphomas, such as marginal zone lymphoma (MZL), mucosa associated lymphoid tissue lymphoma (MALT), and a non-Hodgkin lymphoma. There were also reports of IgG4-RD concurrent with pancreatic cancer and colon carcinoma (87).

To summarize, increased serum levels of IgG4 may in some instances may be useful in the differentiation of clinical conditions. Maślińska et al. reported (69) the lowering of serum concentration of IgG4 below the established normal range in patients with pSS and suggested that it may be also a marker of this autoimmune disease. It was pointed out that C4 hypocomplementemia found in a significant number of pSS patients may have an impact on the reduction of IgG4 concentration and is also an indicator of disease activity taken into account in the ESSDAI assessment (88).

There is also an increase in the concentration of IgG4 in chronic inflammation, which takes place, for example, in active RA (89). It can be assumed that such an increase is important as an expression of the organism’s attempt to inhibit the inflammatory process, with IgG4 playing a positive role as an inhibitory antibody (4). Some authors also concluded that the elevation of IgG4 serum levels in RA concerns a distinct subtype of patients RA with a worse course of the disease and a poorer response to the treatment with classical disease-modifying drugs (cDMARDs) such as methotrexate and leflunomide (90). Umekita et al. (91) described the case with arthropathy with infiltrate IgG4-positive plasma cells in the synovium without any other organ involvement, and the authors argued that the image did not correspond to RA. This topic seems debatable. In the IgG4 RD, arthritis with synovial hyperplasia and infiltration of IgG4+ plasma cells may occur.

An interesting work was recently published by Olejarz et al. (92) showing that in Graves’ orbitopathy (GO), the serum concentration of IgG4 is increased, especially in patients diagnosed at a younger age and with a more severe course of GO with a higher Clinical Activity Score (CAS). The authors suggested that the diagnosis of GO with elevated IgG4 serum concentration in patients with previously established diagnosis of Graves’ disease, as well as the differential diagnosis of conditions such as IgG4 RD or overlapping syndrome, should be taken into account.

From the clinical point of view, other diseases located in the eyeball and the periocular area such as orbital pseudotumor, orbital neoplasms (malignant as lymphomas or benign), infections, orbital myositis, sarcoidosis, and inflammatory orbitopathy in the course of vasculitides especially granulomatosis with polyangiitis (GPA) should be considered in the differential diagnosis. The above considerations indicate the scale of difficulties in the proper diagnosis of IgG4-RD in everyday clinical practice.

IgG4 antibodies found a place in immunotherapy, as their specific anti-inflammatory and protective abilities make them an advantageous subclass in the design of therapeutic antibodies, especially when there is a need to block other antibodies. The interesting therapeutical option is a pembrolizumab-humanized anti-receptor monoclonal antibody programmed cell death-1 (PD-1) which consists of IgG4/kappa isotype with stabilizing modification of the Fc region sequence. This monoclonal antibody found use in therapy of several neoplasms, among others melanoma, cervical cancer, esophageal cancer, colorectal cancer, and triple-negative breast cancer) (93). In June 2020, the Food and Drug Administration (FDA) granted accelerated approval to pembrolizumab for the treatment of patients with unresectable or metastatic tumor mutational burden-high solid tumors, which have progressed following prior treatment and who have no satisfactory alternative treatment options (94). This approval concerns both adult and pediatric patients.

Another interesting drug is nivolumab—a fully human immunoglobulin G4 and a PD-1 immune checkpoint inhibitor antibody. It has demonstrated efficacy in the treatment of patients with advanced non-small-cell lung cancer (NSCLC) as well as melanoma, urothelial cancer, renal cell carcinoma (RCC), or malignant pleural mesothelioma (MPM) (95). Nivolumab was also approved by FDA for the treatment of patients with completely resected esophageal or gastroesophageal junction (GEJ) cancer with residual pathologic disease, who have received neoadjuvant chemoradiotherapy, as well as for patients with metastatic gastric cancer and esophageal adenocarcinoma in combination with chemotherapy (96). The efficacy of nivolumab (anti-PD-1) in combination with ipilimumab (anti-CTLA-4) in the treatment of melanoma or metastatic or recurrent non-small cell lung cancer (NSCLC), in the latter case also with platinum-doublet chemotherapy, has also been demonstrated (97).

The disbalance between IgG4 and other classes or subclasses of immunoglobulins, depending on how it is shaped and in which disease it occurs, may lead to various outcomes tilting the clinical picture toward inhibition of inflammation or its activation, e.g., in allergic diseases where the IgG4 role is strongly inhibitory and protective, in IgG4 RD where IgG4 is an indicator of inflammation and uncontrolled fibrosis (however, the overall role of IgG4 *per se* in IgG-RD is unclear) and in neoplasms, in which the pathogenic role of IgG4 is overwhelming.

Particularly, the interest in their importance in IgG4-related diseases has increased, but no specific IgG4 antibodies have been identified in them, and in the affected organs, infiltrates are composed of many different cells apart from plasma cells that produce this immunoglobulin, and not all patients also present high levels of IgG4 in the serum.

## Concluding Remarks

Immunoglobulin G4 may play both pathogenic and protective role (as a blocking antibody with anti-inflammatory properties) in the human body and disbalance between this and other immunoglobulins may be, depending on circumstances, interpreted in different ways, e.g., in malignances and allergic diseases. The elevation of IgG4 levels by itself is not always an indication of an ongoing pathologic process, as the role of IgG4 is very diverse. The unique properties of this immunoglobulin, such as FAE and its influence on the other immunoglobulins, most likely determine such a variability of its impact. The mere increase in the concentration of IgG4 in the patient’s serum without taking into account all the accompanying phenomena and tests, including histopathological ones, cannot form the basis for the diagnosis of a specific disease. The recent establishment of IgG4-RD has brought IgG4 into the spotlight and raised interest in its involvement in rheumatic and autoimmune diseases. As we learn more about IgG4, the need for taking its presence into consideration during diagnostic and therapeutic process will grow.

## Author Contributions

MM—first author, author for correspondence, concept of the work, writing, literature review, final acceptance. JD-C—writing, literature review, final acceptance. MJ—writing, literature review, final acceptance.

## Conflict of Interest

The authors declare that the research was conducted in the absence of any commercial or financial relationships that could be construed as a potential conflict of interest.

## Publisher’s Note

All claims expressed in this article are solely those of the authors and do not necessarily represent those of their affiliated organizations, or those of the publisher, the editors and the reviewers. Any product that may be evaluated in this article, or claim that may be made by its manufacturer, is not guaranteed or endorsed by the publisher.

## Abbreviations

ANCA, anti-neutrophilic cytoplasmic antibodies; c-ANCA, cytoplasmic anti-neutrophilic cytoplasmic antibodies; p-ANCA, perinuclear anti-neutrophilic cytoplasmic antibodies; CIDP, demyelinating polyradiculoneuropathy; CH, constant domain; FAE, fab arm exchange; FcγR, Fc gamma receptor; VL, light chain variable domain; VH, heavy chain variable domain; IL, interleukin; IgG4 ARD, immunoglobulin G4 autoimmune-related disease; IgG4 RD, immunoglobulin G4-related disease; MuSK-MG, muscle-specific kinase myasthenia gravis; TTP, thrombotic thrombocytopenic purpura; EGPA, eosinophilic granulomatosis with polyangiitis; GPA, granulomatosis with polyangitis; EGPA, eosinophilic granulomatosis with polyangiitis.
